# Using smartphone pupillometer application to measure pupil size and light reflex: An unsuccessful prototype and analysis of the causes of failure

**DOI:** 10.1097/MD.0000000000041682

**Published:** 2025-02-28

**Authors:** Hung-Hsi Tan, Kuo-Chang Lee, Yi-Rong Chen, Yu-Chin Huang, Rih-Shen Ke, Gwo-Jiun Horng, Kuo-Tai Chen

**Affiliations:** a Emergency Department, Chi-Mei Medical Center, Tainan, Taiwan; b Department of Biotechnology, Southern Taiwan University of Science and Technology, Tainan, Taiwan.

**Keywords:** penlight, pupil, pupillary light reflex, pupillometer, smartphone

## Abstract

The accurate assessment of pupillary light reflex (PLR) is essential for monitoring critically ill patients, particularly those with traumatic brain injury or stroke and those in postoperative care. Smartphone-based pupillometers represent a potentially cost-effective solution for addressing this need. We developed a smartphone pupillometer application (app) and evaluated its effectiveness against the penlight test and quantitative pupillometry. This study included 50 volunteers aged >20 years and excluded individuals with neurologic or ophthalmic conditions. The app captured pupillary images by displaying a red circle on the screen, and an algorithm processed these images to calculate the pupil constriction percentage (PCP). The results revealed that the smartphone app often required multiple attempts for successful image acquisition. The obtained PCPs were consistently smaller and less variable than those obtained using the penlight test and a commercial pupillometer (app vs penlight for the right eye: 27.0% [27.0%–8.0%] vs 33.0% [32.3%–39.3%]; app vs pupillometer for the right eye: 27.0% [27.0%–28.0%] vs 35.0% [31.8%–38.3%]; app vs penlight for the left eye: 29.0% [28.0%–29.0%] vs 33.0% [29.8%–34.3%]; app vs pupillometer for the left eye: 29.0% [28.0%–29.0%] vs 36.0% [30.8%–38.0%]; *P* <.001 for all). Notably, the penlight and the pupillometer exhibited comparable PCPs (right eye: penlight vs pupillometer: 33.0% [32.3%–39.3%] vs 35.0% [31.8%–38.3%], *P* = .469; left eye: penlight vs pupillometer: 33.0% [29.8%–34.3%] vs 36.0% [30.8%–38.0%], *P* = .148). The app requires further refinement to yield results comparable to those of established methods. Future iterations can include alternative measurement strategies and dynamic assessment. Penlight and quantitative pupillometry remain indispensable as established tools for PLR.

## 1. Introduction

In the critical care setting, meticulous monitoring of neurologic status is paramount, particularly for individuals with increased intracranial pressure. These patients frequently experience profound sedation or rapid neurologic decline due to conditions such as traumatic brain injury, acute stroke, postoperative neurosurgery, or intracranial masses or post-resuscitation care following cardiac arrest.^[1,2]^ Although a comprehensive neurologic examination provides in-depth insights, it is labor-intensive and ineffective in critically ill patients. Therefore, in conjunction with brain imaging, clinicians often rely on the glasgow coma scale and the pupillary light reflex (PLR) test to measure brainstem function.^[3]^ PLR, which is well-known for detecting brainstem impairment and predicting clinical outcomes in patients with brain injury, has become a cornerstone of neurologic evaluation in critically ill patients.^[4–8]^

Pupillary light reflex is conventionally assessed using a penlight. Although this approach simple and cost-effective, it provides only subjective results. Furthermore, the accuracy of visual evaluations of the pupil is influenced by ambient light conditions, leading to potential disagreements among examiners^[7,9,10]^ These limitations reduce the sensitivity of conventional PLR assessment and, consequently, its applicability to critically ill patients.

Pupillometry, which was first introduced in the 1980s, has gained recognition as a reliable and precise method for measuring PLR.^[11]^ By facilitating quantitative measurements of the pupil size and the speed of pupil constriction, pupillometry facilitates the detection of subtle changes in PLR that may indicate minimal neurologic deterioration.^[9,10]^ This technique also serves to augment the diagnostic approaches for various neurologic conditions, for example, through the early detection of brain herniation in patients with ischemic stroke, prediction of cerebral ischemia after aneurysmal subarachnoid hemorrhage, and assessment of the correlation of intracranial pressure with possibility of elevation in patients with TBI.^[12]^ However, commercial pupillometers are associated with significant costs, lack of portability, and the need for trained operators, limiting their adoption primarily to large academic research centers.^[13,14]^

Smartphone applications (apps) have pervaded the field of medicine because of their widespread use by the general population.^[15]^ Enabled by artificial intelligence trained to recognize image patterns, the utility of smartphones has been demonstrated across medical disciplines.^[16]^ The emergence of smartphone-based pupillometers holds promise, and these pupillometers are poised to address the several limitations of commercial pupillometers.^[16]^ However, despite the availability of select smartphone-based pupillometers, their broad acceptance has been hindered by factors such as high costs, reliance on external wearable components, and a lack of robust academic research.^[13,17–20]^

In this study, we formulated algorithms for pupil identification, which led to the successful development of a prototype smartphone pupillometer app. This app was compared with the standard penlight PLR test and a commercial pupillometer in terms of performance. Our primary objective was to determine the app’s effectiveness for PLR assessment, and we investigated whether it can yield results more precise than those of penlight and closer to commercial pupillometers. Through this smartphone-based approach, we aim to provide a cost-effective, accessible, and user-friendly method for evaluating neurologic status, particularly in critically ill patients.

## 2. Material and methods

### 2.1. Participants

Starting from January 1, 2022, to June 30, 2022, we included 50 volunteers aged > 20 years and excluded pregnant women and individuals with neurologic disorders, acute ophthalmic conditions, or a history of ocular surgery. Two emergency physicians (HHT and KTC) provided thorough explanations of the experimental process to the participants. Baseline data, including information on age and sex, were collected.

This study was approved by the Institutional Review Board of Human Research of Chi-Mei Medical Center, Taiwan (reference: 11010-015), and adhered to the ethical principles of the Declaration of Helsinki. Informed written consent was obtained from all volunteers before their participation in this study.

### 2.2. Introduction of the app

**Figure 1.**
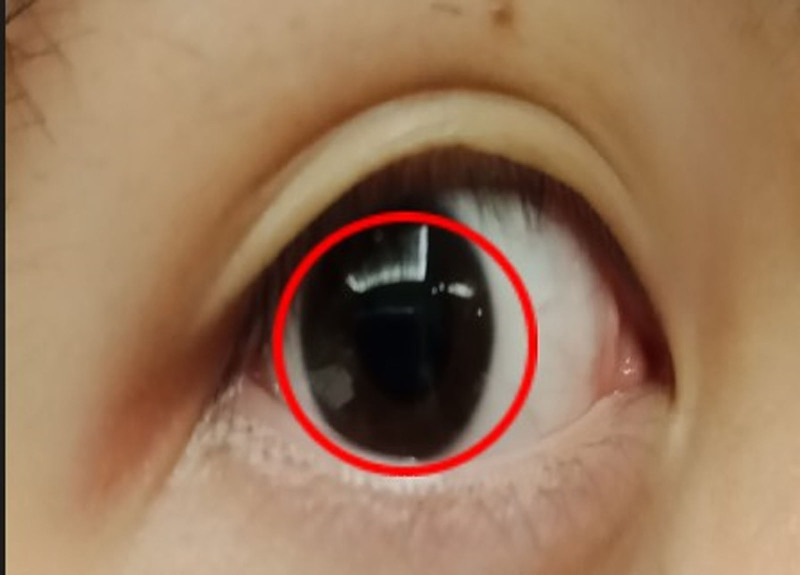
The operation of the application of smartphone pupillometer (app): on the screen of smartphone, the app displayed a red circle on the screen, and the operator adjusted the circle to match the subject’s iris.

The app prototype used in this study was designed using an Android smartphone model (A72; OPPO Mobile Telecommunications Corp., Ltd., GuangDong, China). During the test, the operator held the smartphone close to the participant’s eye; the app screen displayed a red circle. Then, the operator aligned the circle with the participant’s iris (Fig. 1).

Once alignment was achieved, the app captured multiple images in two 2-s periods, separated by a 1-s flash (about 50 photographs during the whole captured process). These images were subsequently saved and transferred to a designated notebook (Vivobook 15; ASUS, Taipei, Taiwan) for further analysis. The current version of this app only tests for a change in pupil diameter using still photograph taken at specific time points.

The algorithm we implemented on the notebook read and converted the recorded images into hue, saturation, value color format. This process was followed by the extraction of color saturation thresholds from the acquired images in addition to the computation of pixel growth rates. If the current growth rate exceeded the previous 1, the pixel extraction process was continued. However, if the current growth rate fell short, the associated threshold value was used as the color saturation threshold, resulting in the generation of a white image outlining the coordinates of the extracted pixels.

After contour identification and pupil diameter filtration, the algorithm focused on the estimation of the pupil diameter. Ultimately, the algorithm provided pupil diameter data, which were used to construct a graphical representation of the changes in the assessed pupil diameter.

### 2.3. Study setup

Each participant underwent a tripartite experimental regimen for each eye. The operator initially conducted the penlight test and documented the results. Subsequently, a pupillometer (NPi-200; NeurOptics, Irvine) was used to assess the participant’s eyes. Next, the operator conducted the app test. In cases where any of the tests failed to produce usable images for estimating the pupil size and pupil constriction percentage (PCP), the test was repeated until a usable image was obtained. A 2-min interval separated the tests to allow the tested eye to return to its baseline condition. Throughout the evaluation period, the participants were instructed to keep their eyes open and stable and to minimize blinking.

### 2.4. Measurement units and data analysis

Data obtained through the penlight and pupillometer tests are presented as millimeters (rounded to the nearest 10th), whereas those obtained through the app test are presented as pixels (rounded to the nearest whole number). Given the variations in measurement units, the PCP was calculated for each test. This percentage was calculated by dividing the degree of pupil constriction after direct illumination by the pupil size before illumination. We then compared the results of the different tests in terms of the PCP and analyzed the intergroup differences with statistical significance.

### 2.5. Safety surveillance

To ensure participant comfort during the experiment, we had previously assessed the app in both indoor and outdoor settings.^[21]^ Notably, the illuminance level in the outdoor environment exceeded that in the indoor environment where the experiment was conducted. To ensure safety, we instructed the participants to promptly report any ocular discomfort resulting from the app test. If any discomfort or adverse effect was reported, we immediately stopped the experiment to prevent further discomfort or harm.

### 2.6. Statistical analysis

Statistical analyses were performed using SPSS (version 15; SPSS Inc., Chicago). Categorical data are presented as the frequency (percentage), whereas continuous data are presented as the median and interquartile range. The differences in the PCP among the tests were analyzed using the Kruskal–Wallis test; this was followed by a post hoc Mann–Whitney *U* test for pairwise comparison. All statistical analyses were 2-tailed. Statistical significance was set at *P* < .05.

## 3. Results

**Figure 2.**
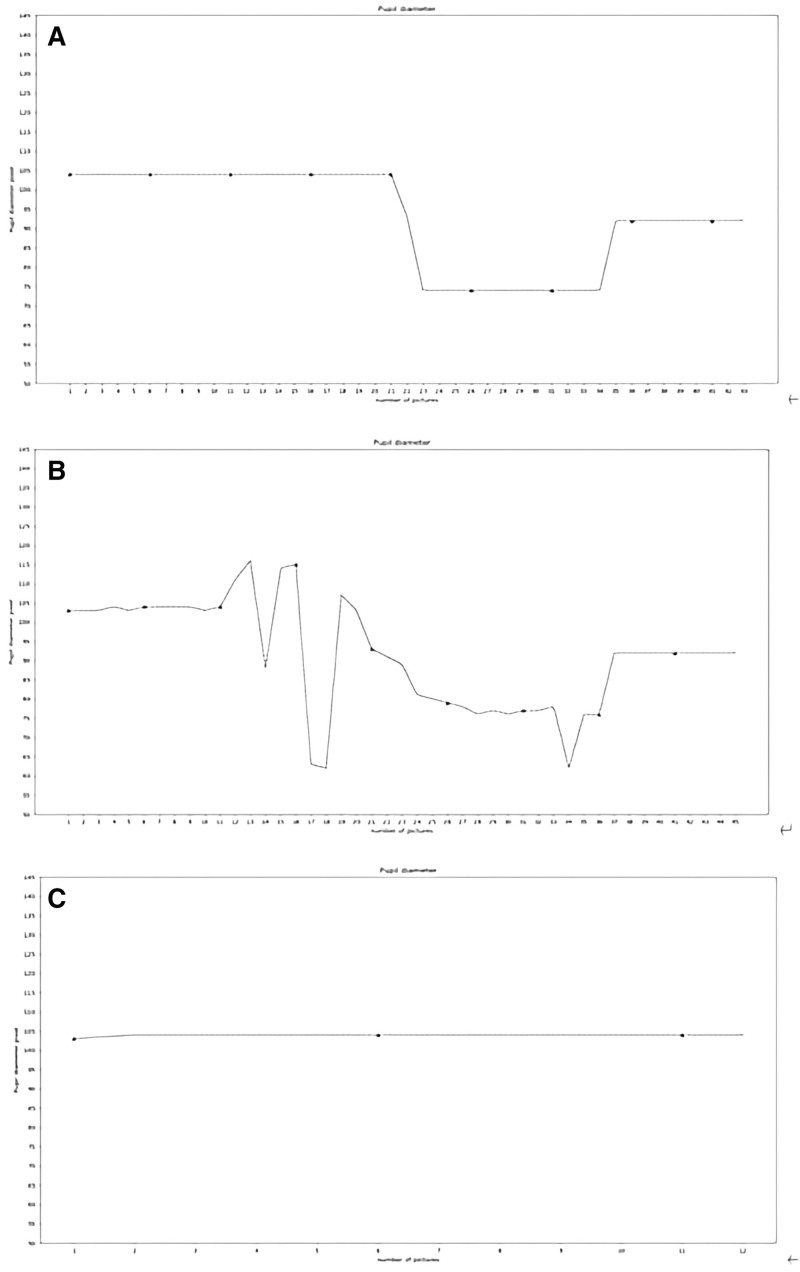
Correct and incorrect graphs obtained in the app. (A) is a correct graph, you can see the original pupil diameter, pupil constriction after light stimulation, and pupil dilation in the recovery period. (B) and (C) are incorrect graphs, they show interruptions in the case of (B) and inaccuracies in the case of (C).

This study included 50 volunteers (median age: 37 [interquartile range: 30–42] years; women: 64%). Both the penlight and pupillometer tests were found to be user-friendly, as the tests conducted using these tools were successfully completed on the first attempt. By contrast, the app test often required multiple attempts to generate images suitable for algorithmic analysis. For successful test execution, 2 and > 3 attempts were required for approximately 30% and 6% of all participants, respectively. Figure 2 presents the graphs depicting both analyzable and unanalyzable data (interruptions in the case of B and inaccuracies in the case of C) used to calculate the pupil size and PCP.

**Figure 3.**
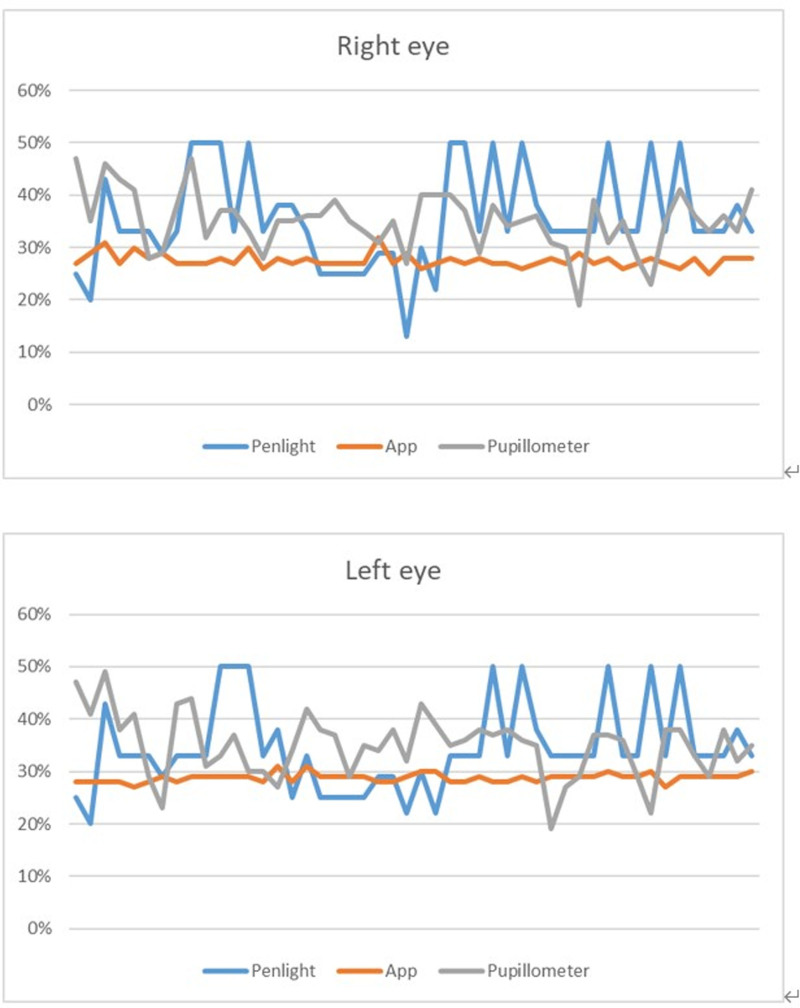
The results of pupil constriction percentages obtained from the 3 instruments for the subjects. The PCPs obtained from the app were significantly smaller compared to those obtained from the penlight and pupillometer. The comparison between the penlight and pupillometer showed statistically insignificant differences. PCPs = pupil constriction percentages.

Figure 3 illustrates the results of PCPs obtained from the 3 instruments for the subjects. Significant differences were observed when comparing the PCPs of the 3 tests (right eye: *P* < .001, left eye: *P* < .001). The PCPs obtained from the App were significantly smaller and less variable compared to those obtained from the penlight and pupillometer (app vs penlight for the right eye: 27.0% [27.0%–8.0%] vs 33.0% [32.3%–39.3%]; app vs pupillometer for the right eye: 27.0% [27.0%–28.0%] vs 35.0% [31.8%–38.3%]; app vs penlight for the left eye: 29.0% [28.0%–29.0%] vs 33.0% [29.8%–34.3%]; app vs pupillometer for the left eye: 29.0% [28.0%–29.0%] vs 36.0% [30.8%–38.0%]; *P* < .001 for all). However, no significant difference was noted in the PCP between the penlight and pupillometer tests (Right eye: penlight vs pupillometer: 33.0% [32.3%–39.3%] vs 35.0% [31.8%–38.3%], *P* = .469; Left eye: penlight vs pupillometer: 33.0% [29.8%–34.3%] vs 36.0% [30.8%–38.0%], *P* = .148). The PCPs obtained through the different tests are presented in Table 1. No participant reported any instance of ocular discomfort throughout this study.

**Table 1:** Pupil constriction percentages.

	Right, median (IQR)	Kruskal–Wallis test	Left, median (IQR)	Kruskal–Wallis test
App	27.0% (27.0%–28.0%)	*P* = .000	29.0% (28.0%–29.0%)	*P* = .000
Penlight	33.0% (32.3%–39.3%)		33.0% (29.8%–34.3%)	
Pupillometer	35.0% (31.8%–38.3%)		36.0% (30.8%–38.0%)	

		Mann–Whitney *U* test		Mann–Whitney *U* test
App vs pupillometer		*P* = .000		*P* = .000
App vs penlight		*P* = .000		*P* = .000
Penlight vs pupillometer		*P* = .469		*P* = .148

IQR = interquartile range.

## 4. Discussion

Quantitative pupillometry, whose validity surpasses that of the penlight test, is widely used in neurologic assessments for the early detection of any neurologic deterioration in patients with TBI. This approach has led to a paradigm shift in examination methods.^[22]^ In the present study, to enhance operational convenience, we developed an easily accessible tool that simulated pupillometry. We meticulously evaluated the performance of this app and compared its performance with that of the penlight and pupillometer tests; the pupillometer served as the benchmark for comparison. However, the results obtained using the newly developed app did not align with those obtained using the pupillometer; this finding indicates the need for further refinement and enhancement. Furthermore, while the penlight results were comparatively favorable, our findings underscored that the present iteration of the App might not equal the efficacy of the traditional penlight approach for skilled practitioners. Additionally, the PLR can be separated into several distinct variables.^[23]^ Simply measured PCP cannot represent details of PLR.

Piaggio et al^[20]^ outlined 5 crucial developmental phases for a smartphone pupillometer: pupil stimulation and video acquisition, preprocessing, image processing, system integration, and technical validation. Consistent with these phases, we plan to analyze these steps for the further enhancement of future versions of the app.

### 4.1. Pupil stimulation and video acquisition

In our study, we used NeurOptics pupillometry as a model. This tool is capable of capturing 30 frames per second over a 3-s recording period for validity assessment.^[5]^ By contrast, our app obtained separate photos for the determination of the smallest pupil size through comparison with previous iterations. This operational mode lacked dynamic veracity and hindered the precise evaluation of the minimum pupil dimension during maximum constriction.

In a previous study, external cardboard support was used to establish the camera-to-eye distance and to optimize focus.^[17,19,24]^ However, in the present study, we refrained from adopting this approach to enhance user experience. Future iterations of the app should explore alternatives for measuring the pupil diameter. The literature proposes 2 alternative approaches. One approach involves the use of the pupil-to-iris ratio, a dimensionless metric less susceptible to variations in the camera-to-patient distance.^[13,25]^ The other approach utilizes the neurological pupil index (NPi) – an algorithmic parameter integrating multiple variables; the use of this parameter facilitates the robust assessment of pupillary reactivity.^[26]^

### 4.2. Preprocessing

In our experiment, red–green–blue images were transformed into the hue, saturation, and value format; subsequent analyses focused solely on the saturation channel.^[20]^ This common technique is dependent on ambient light and duration, which potentially affect photo quality.^[27,28]^ To further enhance image fidelity, future iterations should use infrared camera systems similar to those used in commercial pupillometers.

### 4.3. Image processing

**Figure 4.**
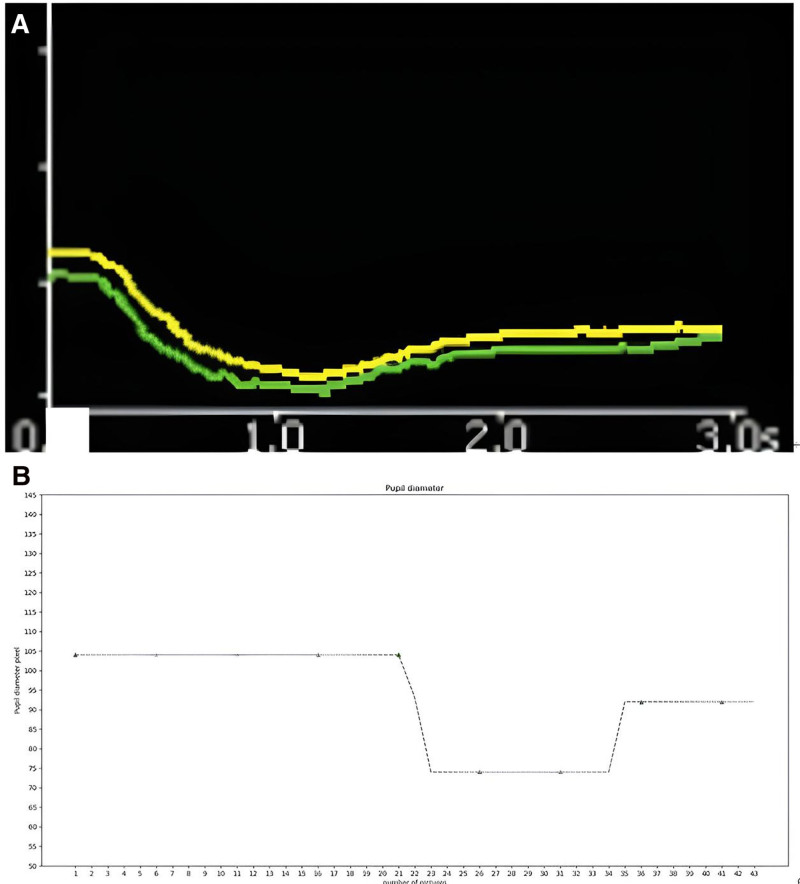
(A) is an image taken from pupillometer, which shows a smooth curve of pupil changes. (B) is an app image: first a flat line, then the graph demonstrates a steep descent followed the light stimulation. After that it shows a sudden increase and remains flat afterward.

The primary limitation of our app was its suboptimal discrimination of pupils, as shown in Figure 4. This abnormal pattern – beginning with a flat line, followed by a steep descent post stimulation and then by a sudden ascent and subsequent flat trajectory – markedly deviates from the smooth curve characterizing pupillometer data. This discrepancy highlights the app’s limitations in accurately discerning, identifying, and measuring pupil size changes.^[6–9]^ Our app’s tendency to identify basic changes resulted in the generation of vertical and flat lines, rather than the nuanced curves generated using pupillometer data – a manifestation of finer differences in the pupil size.

### 4.4. System integration

Directly incorporating the analytical algorithm into the app offers practical streamlining of testing, data analysis, and result presentation processes. Eliminating the need to transmit images for external analyses enhances efficiency and user-friendliness.

### 4.5. Technical validation

Thorough validation across a range of Android-based and iPhone OS-based smartphones is essential to confirm the app’s effectiveness and consistency across devices with varying specifications, which can affect app performance. Rigorous cross-device testing ensures sustained accuracy and reliability.

### 4.6. Device comparisons

The penlight test, despite having obvious interrater discrepancies, can be proficiently used by skilled health-care practitioners for the accurate estimation of the pupil diameter.^[5,29]^ However, the effective application of this test depends on training and experience. By contrast, pupillometers, by transforming qualitative data into quantitative data, facilitate precise PLR assessment. This capability enables the detection of subtle variations in the pupil diameter, thereby facilitating meticulous evaluations of pupillary reactivity. Solari et al^[16]^ demonstrated the potential of quantitative pupillary light reactivity assessments in predicting clinical outcomes after cardiac arrest.

The NPi has emerged as an advanced paradigm, integrating several variables for determining patients’ neurologic status.^[12,16,26–28,30]^ The comprehensive insights provided by the NPi are particularly relevant in critical care settings, where it can facilitate early interventions for critically ill patients.^[8,15]^ Previous smartphone app-based studies, including that by Carrick, have supported the future use of smartphone-based pupillometry.^[31]^ We envision a meticulously designed smartphone pupillometer that can yield results similar to those of conventional pupillometers, supporting the various clinical applications of smartphone pupillometers.

In Table 2, we compared the presented app with 6 studies using smartphone apps to measure pupil size and to detect PLR. Three studies were conducted on the Android system, while the other 4 were on iPhone OS. Additionally, 3 earlier studies required external equipment attached to the smartphone. Currently, there is no universal standard for measurement accuracy, and only two apps are commercially available.

**Table 2:** Comparison of similar studies in the literature.

Studies	System	External equipment	Experimental subjects	Findings	Available app
Kim (2013)^[19]^	Android	Eye Cap	16	Constriction ratios of Pupil are in normal range	No
Calandra (2017)^[18]^	iOS	Headset	30	Pupil measurement: Mean error smaller than 2 pixels	Yes
Mariakakis (2017)^[17]^	iOS	Headset	42	Pupil measurement: median error of 0.30 mm	No
McAnany (2018)^[13]^	iOS	Nil	15	Excellent agreement between app and pupillometry	Yes
Neice (2021)^[25]^	iOS	Nil	13	Pupil measurement: 95% confidence interval of ± 0.26	No
Piaggio (2021)^[20]^	Android	Nil	Not mentioned	Pupil measurement: median error of 0.30 mm	No
Tan (2025)	Android	Nil	50	Less accurate than pupillometry	No

iOS = iPhone OS.

### 4.7. Strengths and limitations of smartphone pupillometer and further utility

A successful smartphone pupillometer app offers standardized and quantitative measurements of a patient’s PLR. These data can aid in neurological assessments, early detection of intracranial hypertension, and prognostication of acute brain injuries.^[5]^ Furthermore, data from the app can provide valuable insights into perception, language processing, memory, decision-making, emotions, and cognitive development.^[32]^

From the analysis of images taken from pupillometry, eye saccade patterns, nystagmus, and PLR provide important features for diagnosing vestibular disorders, movement disorders, and optic neuropathies.^[33–35]^ Table 3 summarizes the findings of previous studies. If a smartphone pupillometer app proves to be as effective as pupillometry, further applications of this technique can be explored.

**Table 3:** Literature regarding the utility of pupillometry in translating eye saccade patterns, nystagmus, and pupillary light reflex into clinical diagnoses.

Diagnosis	Presentations on pupillometry	Findings
Vestibular disorder	Nystagmus	Produce the discriminant features for each vestibular disease
Parkinson disease	Saccade patterns/ nystagmus	Saccadic hypometria and jerk nystagmus
Multiple system atrophy	Saccade patterns/ nystagmus	Saccadic hypometria, optokinetic and Jerk nystagmus
Progressive supranuclear palsy	Saccade patterns/ nystagmus	Saccadic hypometria, optokinetic and Jerk nystagmus
Corticobasal syndrome	Saccade patterns/ nystagmus	Saccadic hypometria, optokinetic and Jerk nystagmus
Unilateral optic neuropathy	Pupillary light reflex	Median pupil constriction of the affected eye was 38% smaller than their fellow eye

## 5. Conclusion

Our exploration into smartphone-based pupillometry led to a prototype with a performance that was not on par with that of the established pupillometer. The transition to a new technology in clinical practice is inevitably marked by a gestation period dependent on various factors. Nonetheless, the latent value of precision measurements relies on clinicians’ adept interpretation and exploration. Therefore, our smartphone pupillometer requires further refinement, centering on improvements in pupil stimulation and video acquisition, image processing, and system integration. As we strive to unlock the full potential of this innovation, ongoing collaboration between technology and clinical expertise is the key to realizing this latent promising technology.

## Acknowledgments

This work was supported by the “Allied Advanced Intelligent Biomedical Research Center, STUST” from Higher Education Sprout Project, Ministry of Education, Taiwan.

## Author contributions

**Conceptualization:** Kuo-Tai Chen.

**Data curation:** Hung-Hsi Tan, Kuo-Tai Chen.

**Formal analysis:** Kuo-Chang Lee.

**Investigation:** Yi-Rong Chen.

**Methodology:** Yu-Chin Huang.

**Project administration:** Gwo-Jiun Horng.

**Software:** Rih-Shen Ke.

**Writing** – **original draft:** Hung-Hsi Tan.

**Writing** – **review & editing:** Kuo-Chang Lee, Kuo-Tai Chen.
